# A new species of *Indoganodes* Selvakumar, Sivaramakrishnan & Jacobus, 2014 (Ephemeroptera, Teloganodidae) from Sri Lanka

**DOI:** 10.3897/zookeys.969.56025

**Published:** 2020-09-17

**Authors:** Alexander V. Martynov, Dmitry M. Palatov

**Affiliations:** 1 National Museum of Natural History, National Academy of Sciences of Ukraine, Bohdan Khmelnytsky str., 15, 01030, Kyiv, Ukraine National Academy of Sciences of Ukraine Kyiv Ukraine; 2 Department of Hydrobiology, Biological Faculty, Moscow State University, Leninskie Gory 1/12, 119234, Moscow, Russian Federation Moscow State University Moscow Russia; 3 A.N. Severtsov Institute of Ecology and Evolution of the Russian Academy of Sciences, Leninskij prosp. 33, 119071, Moscow, Russian Federation Severtsov Institute of Ecology and Evolution of the Russian Academy of Sciences Moscow Russia

**Keywords:** Indomalayan realm, larva, mayflies, Pannota, type material

## Abstract

A new species, *Indoganodes tschertoprudi***sp. nov**. is described from Sri Lanka. The genus *Indoganodes* Selvakumar, Sivaramakrishnan & Jacobus, 2014 was previously known only by one species from the Western Ghats (India). The new species differs from *Indoganodes jobini* Selvakumar, Sivaramakrishnan & Jacobus, 2014 by the number of denticles on the claws, shape of the femora, shape of the chalazae on the femora, absence of any median tubercles on the terga, and presence of posterolateral processes only on segments VI–IX. The diagnosis of *Indoganodes* is also emended. Morphological larval affinities of *Indoganodes* and *Ephemerellina* Lestage, 1924 and the probable origin and diversification of *I. tschertoprudi***sp. nov.** are discussed.

## Introduction

Teloganodidae Allen, 1965 is a relatively small family distributed within the Indomalayan realm and southern part of the Afrotropical realm. Endemism is typical for this family, and the many of species in the family have a restricted distribution. Moreover, the Afrotropical and Indomalayan realms are represented by different genera. Four genera of the family occur in Indomalayan realm: *Teloganodes* Eaton, 1882, *Dudgeodes* Sartori, 2008, *Derlethina* Sartori, 2008, and *Indoganodes* Selvakumar, Sivaramakrishnan & Jacobus, 2014. The family Teloganodidae in the Indomalayan region is currently undergoing a detailed investigation; 22 species and three of four genera mentioned above were described during last 12 years (Sartori et al. 2008; Selvakumar et al. 2014; Anbalagan et al. 2015; Martynov et al. 2016; Garces et al. 2020). The most important progress in the investigation of the group within the region was made by Sartori et al. (2008) who published a revision of Oriental Teloganodidae.

Until now, the genus *Indoganodes* was known only from the Western Ghats (India) and only by the larval stage of the sole species, *Indoganodes jobini* Selvakumar, Sivaramakrishnan & Jacobus, 2014.

In this paper, a new species of *Indoganodes* from Sri Lanka is described based on the larval stage. Detailed observations of the larval features of this new species and its comparison with *I. jobini*, the type species from southern India, allows for the emendation of the generic diagnosis.

## Material and methods

All material were preserved in 80–95% EtOH; some paratypes were mounted with Canada balsam on slides.

Administrative districts and geographical coordinates of localities are given according to Google Earth (http://earth.google.com). Photographs were made using a Canon Power Shot A 630 with Ulab XY-B2T microscope in the National Museum of Natural History, National Academy of Sciences of Ukraine (NMNHNASU) and Leica Z16 APO equipped with Leica DFC450 Digital Camera in the I.I. Schmalhausen Institute of Zoology, National Academy of Sciences of Ukraine. Photographs were subsequently processed with LAS Core 3.8 and Helicon Focus.

The type material now is housed in the NMNHNASU in the collection of first author. The inventory numbers (IN) of slides and samples are *672*, *673* (*Sri1Ingsp*) and *674* (*Sri2Ingsp*).

## Results and discussion

### Taxonomy

#### Indoganodes tschertoprudi sp. nov.

Taxon classificationAnimaliaEphemeropteraTeloganodidae

7521984C-092B-56A7-AB96-C58723AA39C1

http://zoobank.org/1BC57DCC-5D00-4EB8-8878-14230D74CB06

**Figure 1.**
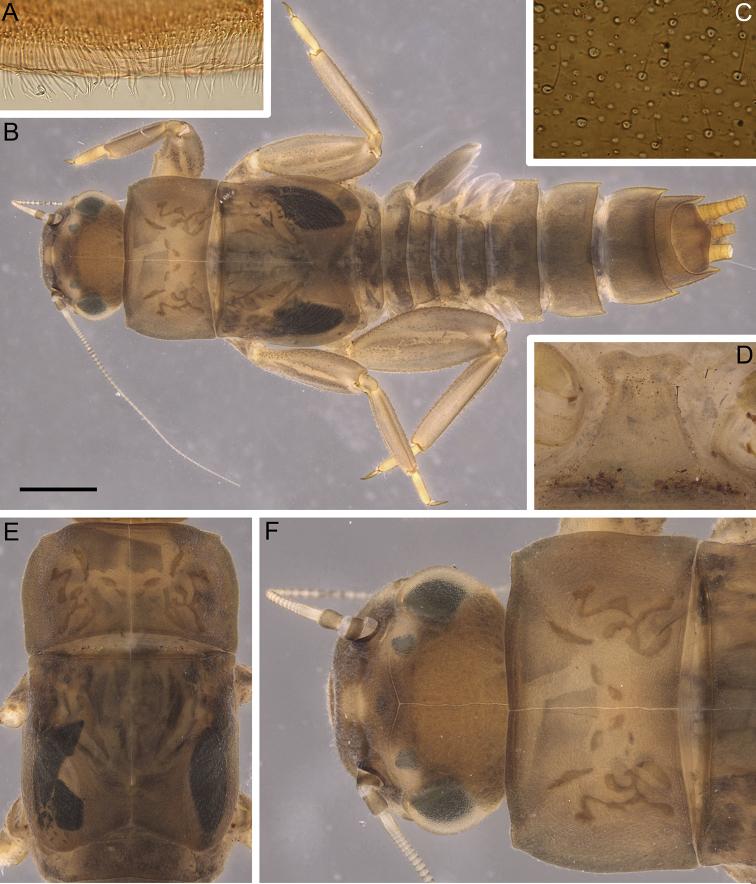
*Indoganodes tschertoprudi* sp. nov., larva, type specimens **A** irregular row of stout setae on anterior margin of clypeus, dorsal view **B** total view of larva **C** head surface **D** prosternum, ventral view **E** thorax, dorsal view **F** head and prothorax, dorsal view. Scale bar: 1 mm.

**Figure 2.**
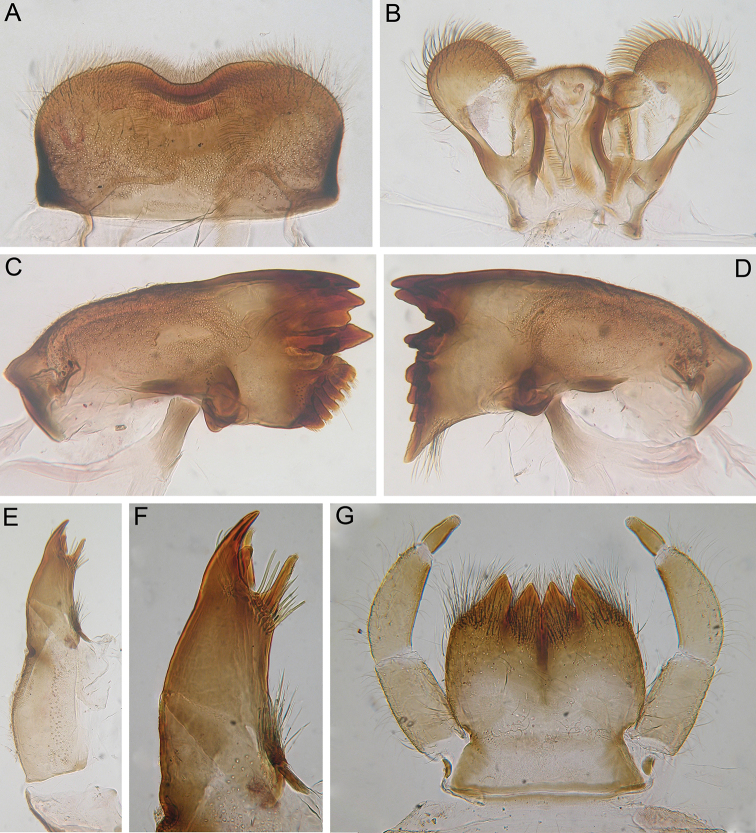
*Indoganodes tschertoprudi* sp. nov., larva, type specimens **A** labrum **B** hypopharynx **C** left mandible **D** right mandible **E** maxilla **F** apical half of maxilla **G** labium.

**Figure 3.**
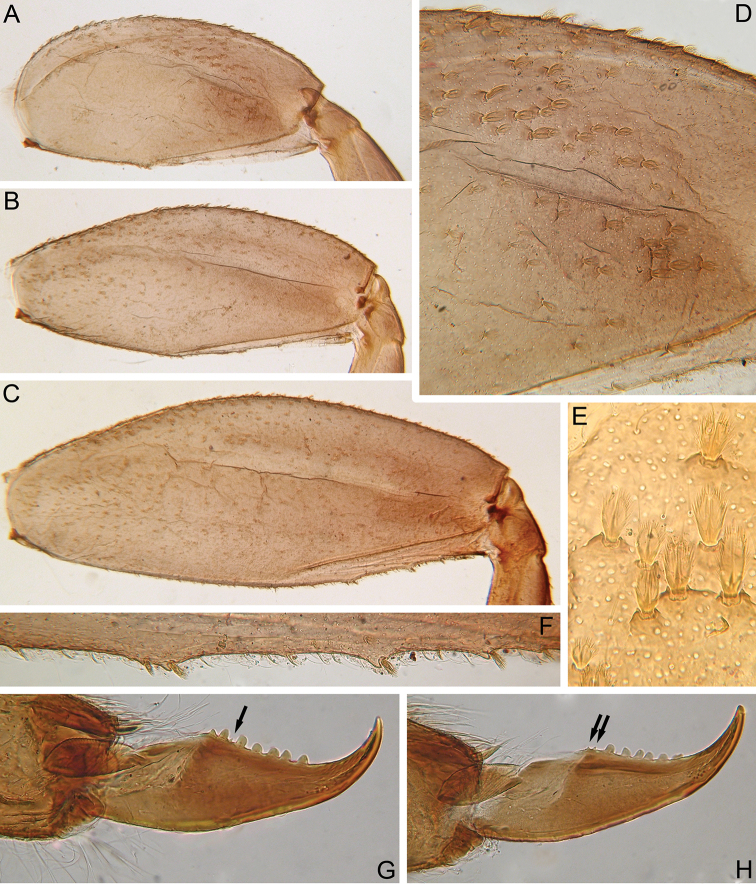
*Indoganodes tschertoprudi* sp. nov., larva, type specimens **A** fore femur, dorsal view **B** middle femur, dorsal view **C** hind femur, dorsal view **D** transversal band of stout setae on fore femur, dorsal view **E** stout setae of dorsal surface of fore femur **F** chalazae with stout setae on inner margin of hind femur **G, H** tarsal claws.

**Figure 4.**
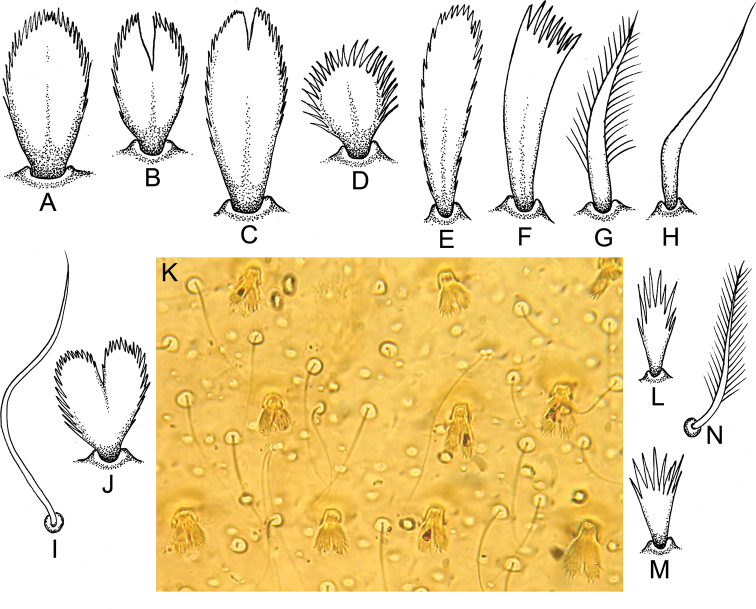
*Indoganodes tschertoprudi* sp. nov., larva, type specimens **A–J** different kinds of stout setae from legs (**A–H, J**) and terga (**I, J**) **K** area of dorsal surface of terga **L–N** setae from surface of sterna.

**Figure 5.**
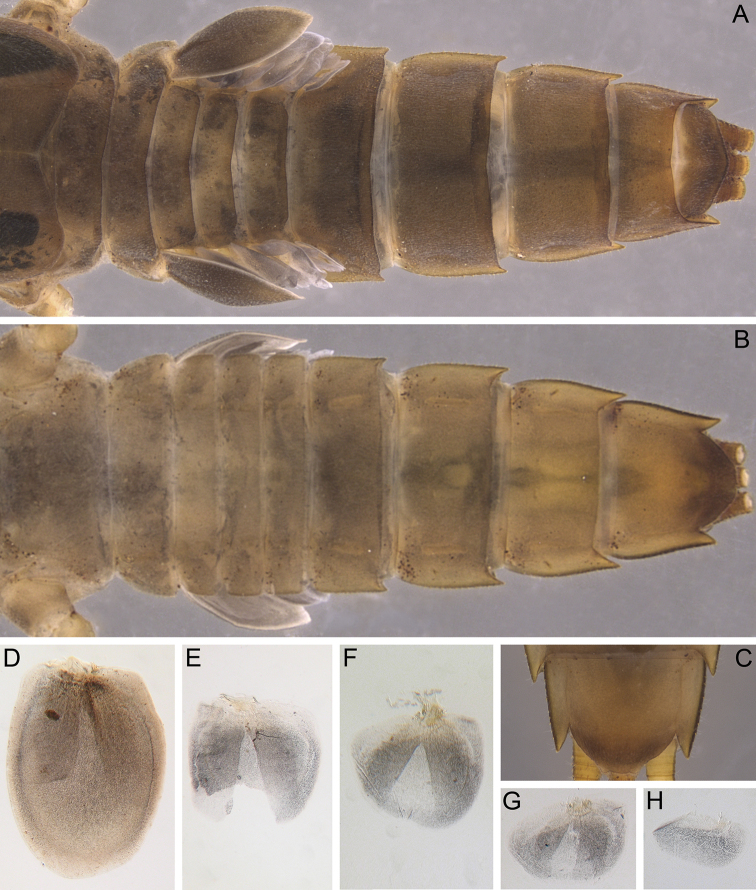
*Indoganodes tschertoprudi* sp. nov., larva, type specimens **A** abdomen, dorsal view **B** abdomen, ventral view **C** sternum IX, ventral view **D–H** gills II–VI.

Figures 1 , 2 , 3 , 4 , 5

##### Material.

***Holotype***: larva (slide 672, mounted with Canada balsam), Sri Lanka, border of Central and Sabaragamuwa provinces, vicinity of Marathenna village, mountain slope, helocrene in valley of large stream, 6.751333, 80.686167, 1390 m a.s.l., Chertoprud M.V. leg., 5.ii.2017 – *IN Sri1Ingsp*. ***Paratypes***: 1 larva (slide 673, mounted with Canada balsam), ibid., Chertoprud M.V. leg., 5.ii.2017 – *IN Sri1Ingsp*. 1 larva (in slide 674 with Euparal), Sri Lanka, Central Province, vicinity of Holmwood Estate, stream (section with almost no current), 6.826389, 80.724444, 1660 m a.s.l., Chertoprud M.V. leg., 4.ii.2017 – *IN Sri2Ingsp*.

##### Etymology.

This species is named after Dr Mikhail V. Chertoprud (Moscow, Russia), who provided the material for this study.

##### Diagnosis.

*Indoganodes tschertoprudi* sp. nov. can be distinguished from the only other known representative of *Indoganodes*, *I. jobini*, by the following combination of characters: (i) tarsal claw with row of 5–8 large, blunt denticles and several (1–3) small, pointed denticles among the large ones (Fig. 3G, H); (ii) several distinct small chalazae bearing stout setae present only in distal part of inner margin of hind femur (Fig. 3C, F); (iii) shape of femora (Fig. 3A–C); (iv) posterolateral processes present only on segments VI–IX, all of them moderately developed (Fig. 5B); (v) posterior margins of all abdominal terga without any median tubercles (Fig. 5A).

##### Description.

**Larva**: body length 8.7–12.5 mm; caudal filaments partially detached, their length ratio to body unknown, paracercus not rudimental. Body light brown (Fig. 1B); head with yellow spots with unclear margins under ocelli; pronotum and mesonotum with several brown smudges (Fig. 1F); legs light brown; ventral side of body dirty yellow to light brown, without any distinct coloration.

***Head*.** Genae small; head without any protuberances; surface of head covered with small hair-like setae and small scale sockets (Fig. 1C); anterior margin of clypeus with dense irregular row of long, stout, hair-like setae with divided apex (Fig. 1A). Labrum (Fig. 2A): wide, anterolateral angles rounded; anterior margin with shallow and wide medial emargination. Dorsal surface (especially anterior part) and anterior margin of labrum densely covered with differently sized (mostly medium-sized and long), thin and stout, hair-like setae. Mostly posterior part of dorsal surface of labrum densely covered with scale sockets. Lateral margins of labrum subparallel, slightly concave. Mandibles (Fig. 2C, D): surface covered with empty scale sockets and scattered short, thin, hair-like setae. Outer margin of mandibles with numerous short and medium-sized hair-like setae. Outer and inner incisors on both mandibles divergent. The molar surface of left mandible composed of three distinct, short, wide ridges; molar surface of right mandible composed of six distinct elongate ridges. Left mandible with bunch of long, hair-like setae under mola; right mandible without setae under mola. Hypopharynx (Fig. 2B): superlinguae with rounded apexes covered by thin and stout, mostly long, hair-like setae; apex of lingua densely covered with short, fine setae. Lingual surface near base with irregular (subparallel to longitudinal axis of body) rows of short, pointed, stout setae (about 18 setae on each side). Maxilla (Fig. 2E, F): palp reduced to small knob, with short, hair-like seta on apex; galea-lacinia with two dentisetae with bristly apexes; galea-lacinia bears one apically rounded, robust denticle on inner margin above dentisetae, ventral surface of maxilla near robust denticle with group of 6 long, stout, hair-like setae; also group of long, stout setae on inner margin near inner dentiseta; base of galea-lacinia with group of long, stout, hair-like setae near inner margin; one or two short or long, pointed, stout setae on dorsal surface near reduced palp. Labium (Fig. 2G): glossae and paraglossae deeply divided, their apexes bluntly pointed, outer margins of paraglossae with deflection. Surfaces of glossae and paraglossae covered with stout and thin, mostly long, hair-like setae; prementum covered with scattered hair-like setae; submentum well developed, covered by same setae and additionally by empty scale sockets. Labial palp 3-segmented; segments II and III robust, but not flattened. Outer margin and adjacent area of dorsal surface of segments I and II covered with long, thin and stout, hair-like setae and several empty scale sockets; several long, hair-like setae on inner margin of segments I and II. Segment III elongated, rounded apically; length/width ratio 2.43–2.75; apex of segment with numerous fine setae, several fine setae present on segment’s surface.

***Thorax*.** Dorsal surface covered with short, thin, hair-like setae and scattered empty scale sockets; tubercles and ridges absent (Fig. 1E). Anterolateral angles of pronotum with small protuberances (Fig. 1F); prosternum without bilobular, spinous process medially (Fig. 1D).

Femora of all legs robust, with longitudinal ridge; outer margin without apical projections (Fig. 3A–C). Fore femur 1.73–2.05 times as long as wide; middle femur 2.27–2.37 times as long as wide; hind femur 2.31–2.58 times as long as wide. Average length ratios of femur, tibia, and tarsus: fore leg 2.13 : 2.24 : 1.00; middle leg 2.78 : 2.85 : 1.00; hind leg 3.48 : 3.51 : 1.00.

Dorsal surface of fore femur with indistinct wide, transversal band of short and medium-sized, oval, stout setae bearing feathered margins and short and medium-sized, feathered, stout setae with divergent margins and cleft at apex (Figs 3A, D, E, 4A–C). Same kind of setae along outer margin and on outer and inner margins (on outer margin, setae more numerous then on inner margin); one stout setae on inner margin on small chalaza. Outer margin of fore femur without chalazae. Entire dorsal surface of fore femur and its margins covered with scattered short, thin, hair-like setae and long, pointed, stout setae with feathered margins.

Ventral surfaces of fore tibia and tarsus with numerous differently shaped, stout setae on inner margin and along it; main types of stout setae are: long, stout setae with feathered margins and pointed apex (Fig. 4G); feathered, stout setae with divergent margins and flat apex (some setae with cleft at apex) (Fig. 4F); medium-sized, stout, hair-like setae (Fig. 4H); elongated, feathered, stout setae with slightly divergent margins and rounded apex (Fig. 4E). Dorsal surface of fore tibia and tarsus covered with medium-sized, hair-like setae; dorsal surface of fore tibia along patella-tibial suture also bears row of differently sized, oval or rounded, feathered, stout setae with cleft at apex in some (Fig. 4A–D). Outer margins of fore tibia and tarsus without stout setae, and only with differently sized, hair-like setae.

Dorsal surfaces of middle and hind femora covered with oval and rounded, medium-sized, feathered, stout setae with cleft at apex in some (Fig. 4B, D), and scattered, short and medium-sized, feathered, stout setae with divergent margins, with cleft at apex (Fig. 4J), and short and medium-sized, oval, stout setae with feathered margins (Fig. 4A–C). Stout setae mostly along outer margin and in central area of femora; stout setae more numerous on outer margins than on inner margins. Additionally, entire dorsal surface and all margins of middle and hind femora covered with a few short, hair-like setae. Middle femur with small, indistinct chalazae bearing stout setae on inner margin (Fig. 3B). Several distinct, small chalazae bearing stout setae present only in distal part of inner margin of hind femur (Fig. 3C, F).

Patella-tibial suture on tibiae of middle and hind legs distinctly shorter than that on fore leg. Setation of middle and hind tibiae and tarsi near that of fore leg, but in contrast to fore leg, outer margins of these tibiae bear short, feathered, oval, stout setae; in immature larvae, row of stout setae more dense and distinct.

Tarsal claw of all legs robust, hooked, its surface covered with several medium-sized, thin, hair-like setae. Claw with row of 5–8 large, blunt denticles and several (1–3) small, pointed denticles among the larger ones (Fig. 3G, H).

***Abdomen*.** All terga without any median tubercles (Fig. 5A). Terga I–X covered with: short, feathered, stout setae with divergent margins and a cleft at apex; mostly short, thin, hair-like setae; empty scale sockets (Fig. 4I–K). Posterior margins of all terga without denticles, only with a few short, thin, hair-like setae. Lateral margins of terga I–V covered only with scattered thin, hair-like setae; lateral margins of segments VI–X also with short, feathered, stout setae. Posterolateral processes presented on segments VI–IX; all of them moderately developed; largest processes on segments VI–IX (Fig. 5B, C). All sterna covered with: short, feathered, thin hair-like setae (Fig. 4N); short setae with divergent, feathered margins, and feathered apex (Fig. 4L, M); empty scale sockets.

Segment I without gills; gills present on abdominal segments II–VI (Fig. 5A). Gill II light brown (Fig. 5D), covered with scattered short, thin, hair-like setae and empty scale sockets; dorsal lamella semi-operculate, without transverse band of weakened membrane, incompletely covers other gills. Ventral lobes of gills II–V bifurcated, multifoliate (Fig. 5D–G); gill VI simple, without medial cleft (Fig. 5H).

Basal part of caudal filaments with feathered, stout setae and stout, hair-like setae at articulations; stout setae shorter, mainly oval on dorsal side of the filaments and elongated on ventral side.

***Winged stages***: unknown.

##### Distribution and biology.

**Figure 6.**
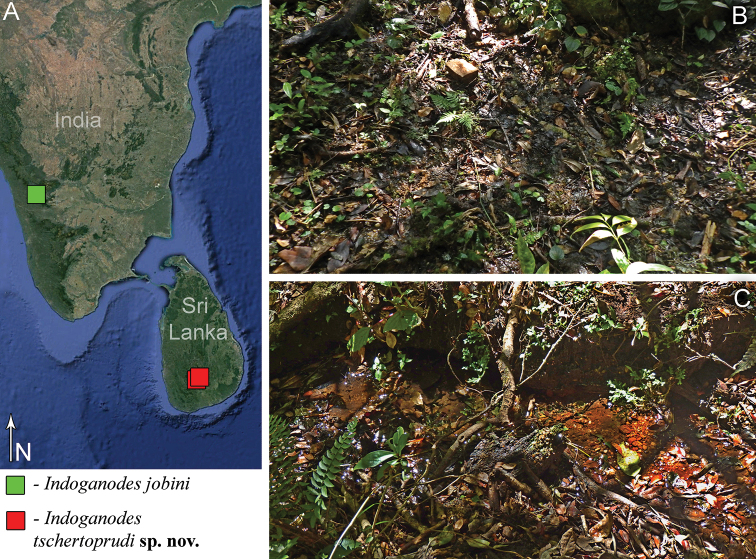
Distribution of *Indoganodes* representatives (**A**), and habitats of *Indoganodes tschertoprudi* sp. nov. (**B, C**) **A** map of genus *Indoganodes* distribution **B** type locality, helocrene spring in valley of large stream, vicinity of Marathenna village, border of Central and Sabaragamuwa Provinces, Sri Lanka (February 2017, photo by M.V. Chertoprud) **C** small stream, section with almost no current, vicinity of Holmwood Estate, Central Province, Sri Lanka (February 2017, photo by M.V. Chertoprud).

Larvae of new species were found in wooded gullies in the mountains of Sri Lanka in the subtropical altitudinal zone (altitude 1390–1660 m a.s.l.) (Fig. 6A). Two larvae of *I. tschertoprudi***sp. nov.** were collected from a helocrene spring in the valley of a large stream (Fig. 6B). The maximum depth of the spring was only 1–2 cm deep; there was no current and the bottom was covered with mud, leaf litter, and detritus. Co-occurring species of mayflies recorded from this habitat were *Ephemera* sp. (Ephemeridae) and *Kimminsula* sp. (Leptophlebiidae). Another larva of this new species was collected along with Ephemera sp. from a small stream, in a section with almost no current and having a muddy bottom (Fig. 6C).

## Discussion

**Figure 7.**
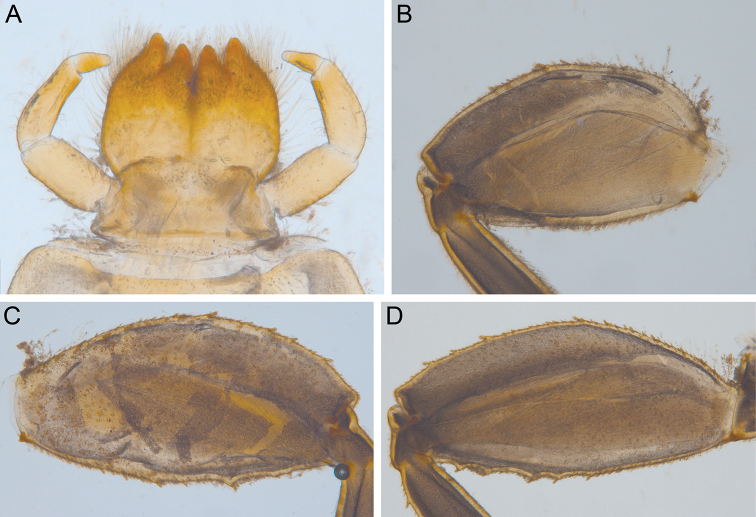
*Indoganodes jobini* Selvakumar, Sivaramakrishnan & Jacobus, 2014, larva, type specimen **A** labium **B** fore femur, dorsal view **C** middle femur, dorsal view **D** hind femur, dorsal view (photos by C Selvakumar).

Selvakumar et al. (2014) established the genus *Indoganodes* based on the larvae of *I. jobini*. According to the original description, this genus is distinguished from other genera of Teloganodidae by the following combination of characters: (i) prosternum without medial bilobular, spinous process; (ii) poorly developed abdominal posterolateral processes on segments I–V and well developed abdominal posterolateral processes on segments VI–IX; (iii) hooked tarsal claw, bearing four small, medial denticles; (iv) labrum subquadrate, approximately twice as broad as long, with short, scattered setae over entire dorsal surface; (v) moderately developed superlinguae of hypopharynx; and (vi) left mandible without medioapicalsetal patch. Several additional figures with distinguishing characters of *I. jobini* are provided in Figure 7A–D.

The new species of *Indoganodes* reveals features that enable us to emend the diagnosis of the genus as follows: characters (i) and (iv–vi) stay unchanged. Emended characters are (ii) abdominal posterolateral processes well developed on segments VI–IX; the processes on segments I–V absent or poorly developed; (iii) claw with one row of denticles, claw with up to eight large denticles and three small denticles, they might alternate in row. Additional characters are: (vii) glossae and paraglossae deeply divided and bluntly pointed; (viii) forefemur not flattened, without distinct and regular, transversal row of stout setae; (ix) outer margin of fore femora without long stout setae; (x) paracercus not reduced.

The Gondwanan origin of the Teloganodidae (McCafferty and Wang 1977) and the close relationship of *Indoganodes* with *Ephemerellina* Lestage 1924 (Selvakumar et al. 2014) apparently hold good, as corroborated by our observations.

The genus *Indoganodes* is most similar to *Ephemerellina* in the combination of some larval characters (winged stages of both *Indoganodes* species are not described): (i) shape of labrum, (ii) fore femur not significantly flattened, (iii) absence of distinct, narrow, transversal row of stout setae on fore femur, (iv) inner margin without a row of setae continuing on dorsal surface near articulation with trochanter, (v) absence of filamentous gill I, (vi) gills present on segments II–VI, (vii) semi-operculate dorsal lobe of gill II, (viii) deep division of glossae and paraglossae, (ix) unreduced paracercus, and some other characters.

*Indoganodes* and *Ephemerellina* are isolated biogeographically, with *Ephemerellina* from the Afrotropical realm and *Indoganodes* from the Indomalayan realm. It is probable that they share a common ancestor from the African continent. After eastern Gondwana, including also India and Sri Lanka, had broken free of Africa about 100 million years ago, these genera evolved separately. Sri Lanka later split from India, and since the Pliocene (5.33–2.58 million years ago), the geographic position of Sri Lanka has been similar to that at present. However, during the periodic low sea levels in the Pleistocene (2.58–0.0117 million years ago), there was a land bridge between India and Sri Lanka, and two-way dispersal of mainly terrestrial fauna was facilitated. The last land bridge was cut off by rising sea levels 5,000–8,000 years ago as the Pleistocene gave way to warmer climates and northern glaciers retreated during the Holocene (Dittus 2017). In our opinion, the morphological proximity of *I. jobini* and *I. tschertoprudi* sp. nov. testify that separation of the species was recent, most probably after the disconnection of India and Sri Lanka at the end of the Pleistocene.

Presently, only the narrow Palk Strait separates Sri Lanka and India. Although mayflies have winged stages capable of dispersal, the teloganodid fauna of the island shares no species with India or other countries of Indian subregion, which is in contrast to the vast number of other mayfly families (Sivaramakrishnan et al. 2009). All species of Teloganodidae found in Sri Lanka are island endemics. These include, in the genus *Teloganodes*, *T. tristis* (Hagen, 1858), *T. insignis* (Wang & McCafferty, 1996), *T. tuberculatus* Sartori, 2008, *T. jacobusi* Sartori, 2008, and *T. hubbardi* Sartori, 2008, and, in the genus *Indoganodes*, *I. tschertoprudi* sp. nov. This teloganodid fauna has no endemic genera. The monotypic genus *Macafertiella* Wang, 1996, which was described from Sri Lanka (Wang and McCafferty 1996), is now considered a junior synonym of *Teloganodes* (Sartori et al. 2008), and the distribution of *Teloganodes* is presently thought to be restricted to southern India (Western and Eastern Ghats) and Sri Lanka (Selvakumar et al. 2014).

## Supplementary Material

XML Treatment for Indoganodes tschertoprudi
